# High intensity intermittent exercise improves cardiac structure and function and reduces liver fat in patients with type 2 diabetes: a randomised controlled trial

**DOI:** 10.1007/s00125-015-3741-2

**Published:** 2015-09-09

**Authors:** Sophie Cassidy, Christian Thoma, Kate Hallsworth, Jehill Parikh, Kieren G. Hollingsworth, Roy Taylor, Djordje G. Jakovljevic, Michael I. Trenell

**Affiliations:** Institute of Cellular Medicine, Newcastle University, Newcastle upon Tyne, NE2 4HH UK; Magnetic Resonance Centre, Campus for Ageing and Vitality, Newcastle University, Newcastle upon Tyne, NE4 5PL UK; UKRC Centre for Ageing and Vitality, Faculty of Medical Sciences, Newcastle University, Newcastle upon Tyne, NE2 4HH UK

**Keywords:** Fatty liver, Heart diseases, Left ventricle, MRI, Type 2 diabetes mellitus

## Abstract

**Aims/hypothesis:**

Cardiac disease remains the leading cause of mortality in type 2 diabetes, yet few strategies to target cardiac dysfunction have been developed. This randomised controlled trial aimed to investigate high intensity intermittent training (HIIT) as a potential therapy to improve cardiac structure and function in type 2 diabetes. The impact of HIIT on liver fat and metabolic control was also investigated.

**Methods:**

Using an online random allocation sequence, 28 patients with type 2 diabetes (metformin and diet controlled) were randomised to 12 weeks of HIIT (*n* = 14) or standard care (*n* = 14). Cardiac structure and function were measured by 3.0 T MRI and tagging. Liver fat was determined by ^1^H-magnetic resonance spectroscopy and glucose control by an OGTT. MRI analysis was performed by an observer blinded to group allocation. All study procedures took place in Newcastle upon Tyne, UK.

**Results:**

Five patients did not complete the study and were therefore excluded from analysis: this left 12 HIIT and 11 control patients for the intention-to-treat analysis. Compared with controls, HIIT improved cardiac structure (left ventricular wall mass 104 ± 17 g to 116 ± 20 g vs 107 ± 25 g to 105 ± 25 g, *p <* 0.05) and systolic function (stroke volume 76 ± 16 ml to 87 ± 19 ml vs 79 ± 14 ml to 75 ± 15 ml, *p <* 0.01). Early diastolic filling rates increased (241 ± 84 ml/s to 299 ± 89 ml/s vs 250 ± 44 ml/s to 251 ± 47 ml/s, *p <* 0.05) and peak torsion decreased (8.1 ± 1.8° to 6.9 ± 1.6° vs 7.1 ± 2.2° to 7.6 ± 1.9°, *p <* 0.05) in the treatment group. Following HIIT, there was a 39% relative reduction in liver fat (*p <* 0.05) and a reduction in HbA_1c_ (7.1 ± 1.0% [54.5 mmol/mol] to 6.8 ± 0.9% [51.3 mmol/mol] vs 7.2 ± 0.5% [54.9 mmol/mol] to 7.4 ± 0.7% [57.0 mmol/mol], *p <* 0.05). Changes in liver fat correlated with changes in HbA_1c_ (*r =* 0.70, *p <* 0.000) and 2 h glucose (*r =* 0.57, *p <* 0.004). No adverse events were recorded.

**Conclusions/interpretation:**

This is the first study to demonstrate improvements in cardiac structure and function, along with the greatest reduction in liver fat, to be recorded following an exercise intervention in type 2 diabetes. HIIT should be considered by clinical care teams as a therapy to improve cardiometabolic risk in patients with type 2 diabetes.

***Trial registration*:**

www.isrctn.com 78698481

***Funding*:**

Medical Research Council.

## Introductioṅ

Heart disease is the leading cause of morbidity and mortality in type 2 diabetes [1], and more than a quarter of all hospital admissions for heart failure in the West involve a patient with diabetes [2, 3]. Early changes in left ventricular structure and function have been identified in adults with type 2 diabetes prior to any overt cardiac disease. These include pathological hypertrophy [4], reduced end-diastolic blood volume [5], diastolic and systolic dysfunction [5–7], and alterations in strain patterns [6, 8], as identified using sensitive magnetic resonance techniques. MRI measurement has been shown to have greater reproducibility than two-dimensional echocardiography in healthy and failing hearts, while avoiding the ionising radiation exposure associated with computed tomography methods, permitting ethical longitudinal studies [9]. Despite clear cardiac dysfunction in type 2 diabetes, therapies to target these preclinical cardiac changes are sparse.

Treatment algorithms for type 2 diabetes support a physically active lifestyle at every stage of treatment [10]. Indeed, aerobic and resistance exercises have known benefits to cardiovascular function [11], although little is known about the impacts on cardiac structure and function. More recently, attention has been given to the intensity of exercise, with high intensity intermittent training (HIIT) fast becoming a popular alternative to continuous moderate exercise [12]. HIIT refers to brief intervals of vigorous activity interspersed with periods of low activity or rest, and elicits a strong cardiac response compared with moderate continuous exercise [12, 13]. It therefore has the potential to improve cardiac structure and function in type 2 diabetes. However, the cardiac benefits of HIIT in type 2 diabetes are still to be determined, and the metabolic effects are also unclear. Only two 12 week HIIT studies have shown improvements in HbA_1c_ [14, 15], and the impact of HIIT on ectopic fat (which plays an important role in glucose homeostasis [16, 17]) has not been investigated. We therefore aimed to define the effect of HIIT on cardiac structure and function, regional fat deposition, and glycaemic control in people with type 2 diabetes. We hypothesised that HIIT would improve cardiac structure and function, alongside improving glycaemic control and reducing ectopic fat, in adults with type 2 diabetes.

## Methods

### Patients

**Fig. 1:**
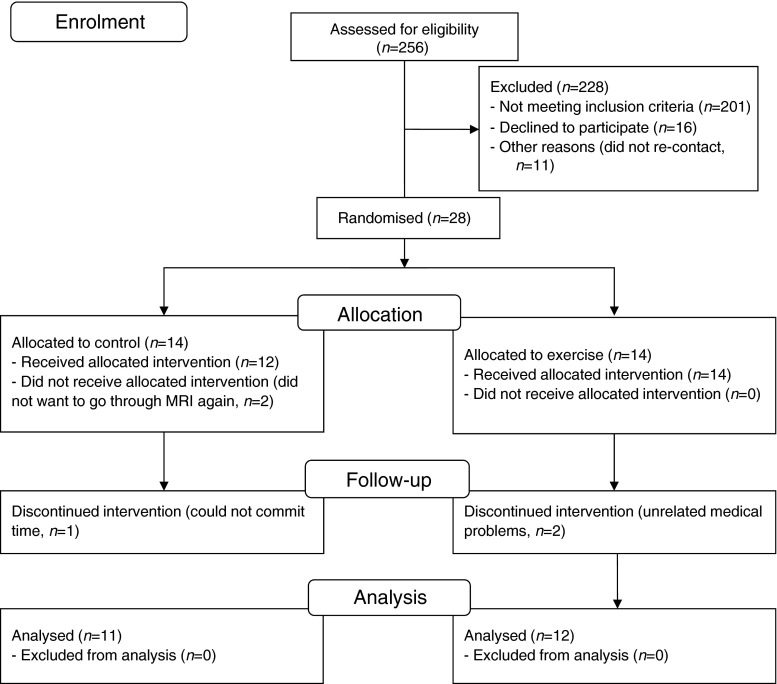
Consort flow diagram showing patient numbers at each stage of the trial

Type 2 diabetic patients (stable control with diet and/or metformin for at least 6 months) were randomised to a HIIT (*n* = 14) or a control group (*n* = 14). Participant characteristics can be seen in Table 1. Participants were excluded if they had any overt cardiac disease, took part in regular exercise (≥60 min moderate–vigorous activity per week), were being treated with β-blocker medication or had any contraindications to exercise stress testing according to guidelines [18]. The study was approved by the Newcastle and Northeast Tyneside Local Research Ethics Committee and all participants provided written informed consent. Participants were recruited via advertising in local newspapers and diabetes community groups between September 2012 and September 2013. During the study, two participants left for unrelated medical reasons, one participant could not commit the time and two failed to comply with MRI procedures, leaving 12 in the HIIT group and 11 in the control group (Fig. 1).

**Table 1:** Participant characteristics

Characteristic	Control group	HIIT group	*p* value
*n* (men/women)	8/3	10/2	
Age (years)	59 ± 9	61 ± 9	0.70
Time since diagnosis (years)	4 ± 2	5 ± 3	
BMI (kg/m^2^)	32 ± 6	31 ± 5	0.71
Height (cm)	169 ± 9	171 ± 8	0.71
Weight (kg)	90 ± 9	90 ± 15	0.95
HbA_1c_ (%)	7 ± 0.5	7 ± 1	0.87
HbA_1c_ (mmol/mol)	55 ± 6	54 ± 11	0.88
Fasting glucose (mmol/l)	7.0 ± 1.0	6.8 ± 1.6	0.69
2 h glucose (mmol/l)	11.7 ± 3.1	12.5 ± 3.1	0.57
Liver fat (%)	7.1 ± 6.8	6.9 ± 6.9	0.94
$\dot{V}{\mathrm{O}}_{2\mathrm{peak}}$ (ml kg^−1^ min^−1^)	20.3 ± 6.1	21.8 ± 5.4	0.54
Medications			
Metformin	7	7	
Statins	6	7	
BP	5	3	

$\dot{V}{\mathrm{O}}_{2\mathrm{peak}}$ is normalised to body mass

### Experimental protocol and randomisation

Following an initial screening visit, cardiac structure and function, liver and visceral fat, body composition, glycaemic control and blood variables were measured at baseline and after 12 weeks of HIIT or continued standard care. Glucose control was measured within 48–72 h of the final exercise session to control for the acute effect of exercise on glucose uptake. Patients were randomised into groups using a simple random allocation sequence (www.randomization.com). Concealed envelopes with consecutive numbers were locked in a drawer and withdrawn in numerical order by the main author (SC).

### Screening

To determine underlying cardiac disease, a medical history, physical examination, and resting and exercise 12-lead electrocardiography (Custo med, Ottobrunn, Germany) were performed. During the exercise test, gas exchange was measured to determine peak oxygen consumption ($\dot{V}{\mathrm{O}}_{2\mathrm{peak}}$) normalised to body mass. $\dot{V}{\mathrm{O}}_{2\mathrm{peak}}$ was determined as the point at which participants reached volitional exhaustion, participants could no longer maintain a cycling rate of 60 rev/min, or continuing exercise was contraindicated [18]. The test was performed using an electronically braked semirecumbent cycle ergometer (Corival Lode, Groningen, The Netherlands) and resistance was increased by 1 W every 6 s. Before the exercise test, patients completed a 20 min resting period in which they lay supine while beat-to-beat BP was measured by the vascular unloading technique [19].

### Cardiac MRI

**Fig. 2:**
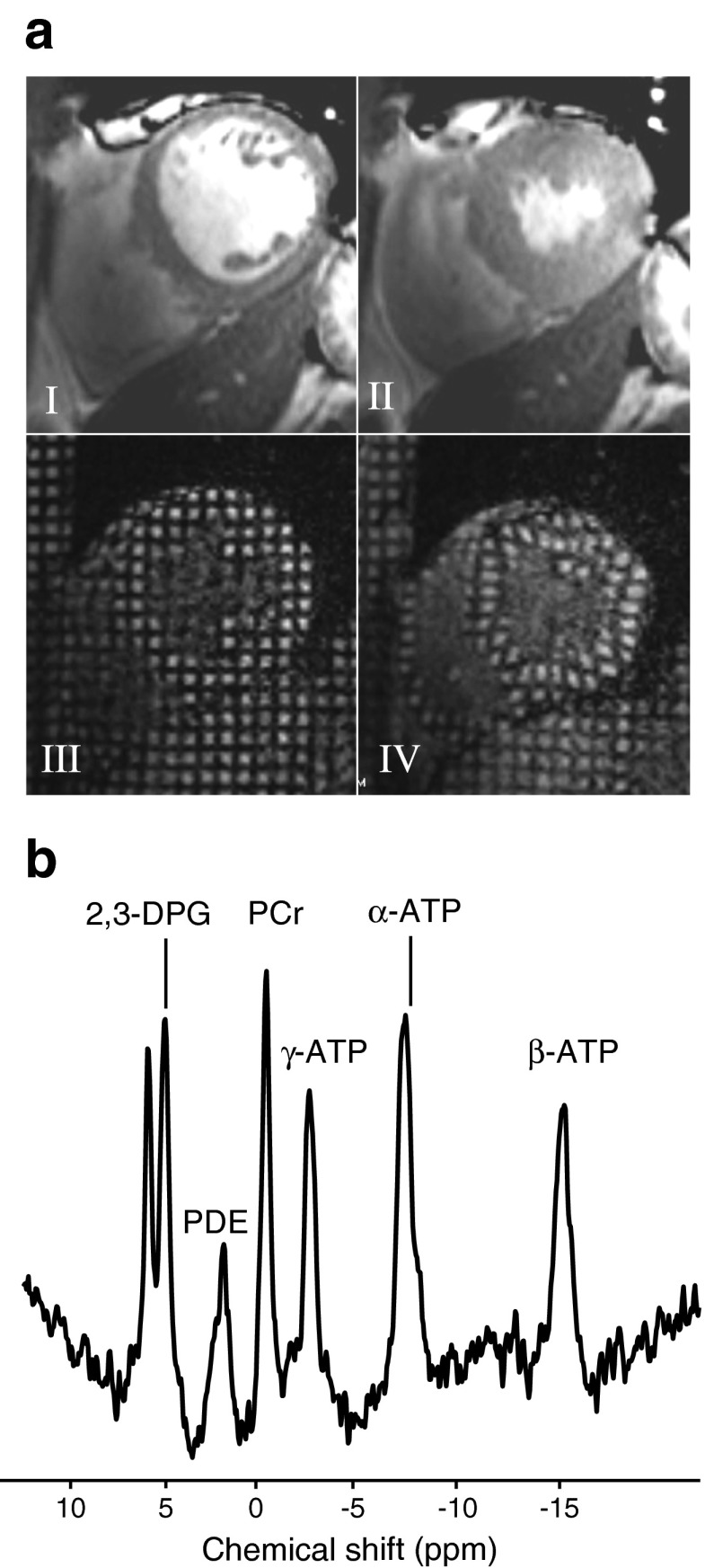
Cardiac MRI techniques. These include (**a**) Cardiac cine imaging (I, II) and cardiac tagging (III, IV) at diastole (I, III) and systole (II, IV), showing how a rectangular grid of nulled signal applied at diastole remains with the tissue through the cardiac cycle, allowing calculation of strain and torsion. (**b**) Phosphorus spectroscopy. Spectrum presented before correction for saturation due to blood content, flip angle at the cardiac tissue and heart rate. DPG, diphosphoglycerate; PDE, phosphodiesters

All examinations were performed using the 3.0 T Philips Achieva MRI scanner with a six channel cardiac array (Philips Medical Systems, Best, The Netherlands). During breath holding, a stack of balanced steady-state free precession images were obtained in the short axis view, covering the entire left ventricle (field of view [FOV] = 350 mm, repetition time [TR] / echo time [TE] = 3.7/1.9 ms, turbo factor 17, flip angle 40°, slice thickness 8 mm, 0 mm gap, 14 slices, 25 phases, resolution 1.84 × 1.37 mm with zero filling to 1.37 × 1.37 mm and temporal duration approximately 40 ms per phase, dependent on heart rate). Using a Viewforum workstation (Philips Medical Systems), the short axis slices at end-diastole and end-systole were used (Fig. 2a) to manually trace endocardial and epicardial borders, with papillary muscles excluded from volume calculations but included in calculations of left ventricular mass. The apical slice was defined as the last slice showing inter-cavity blood pools, and the basal slice as the last slice in which at least 50% of the blood volume was surrounded by myocardium. The analysis was performed by a single observer blinded to group allocation. Details of the algorithm for contour selection and calculation of left ventricular mass, systolic and diastolic variables have been previously published [20]. The eccentricity ratio is a measure of concentric remodelling, and was calculated by dividing left ventricular mass by end-diastolic blood volume. Longitudinal shortening was determined from cine MRI in the four-chamber view by determining the perpendicular distance from the plane of the mitral valve to the apex in systole and diastole.

### Cardiac tagging

Tagged short axis images were acquired. Cardiac tagging works by applying radiofrequency pulses to cancel the MR signal from the myocardium in diastole in a rectangular grid pattern and tracking the deformation of these tags through the rest of the cardiac cycle (Fig. 2a). A turbo-field echo sequence with acceleration factor 9 was used (TR = 4.9, TE = 3.1, flip angle = 10°, number of excitations = 1, SENSE factor 2, FOV 350 × 350 mm, voxel size 1.37 × 1.37 mm with no zero filling and an orthogonal complementary spatial modulation of magnetistion grid with tag spacing of 7 mm). Short axis slices of 10 mm thickness were prescribed. The Cardiac Image Modelling package (University of Auckland, New Zealand) was used to analyse the tagging data by aligning a mesh on the tags between the endo- and epicardial contours. Details of the calculation of strain and torsion variables have been previously published [21].

### Cardiac spectroscopy

Cardiac high-energy phosphate metabolism was assessed using ^31^P-magnetic resonance spectroscopy (see Fig. 2b for an example cardiac spectrum). Cardiac acquisition was vectorcardiogram-gated with the participant in a prone position during free breathing, with a 10 cm diameter ^31^P surface coil used for transmission/reception (PulseTeq, Chobham, UK) and the scanner body coil used for localising images. A cardiac gated one-dimensional chemical shift imaging sequence was used, and to eliminate contamination from liver, a 7 cm slice was selected in the foot–head direction using a selective pulse (of the ‘spredrex’ type, as outlined in [22]). The flip angle subtended at the myocardium was fixed to achieve 50° excitation at the target depth and measured using a variable flip angle sequence on a gadolinium-doped 20 mmol/l phenyl phosphonic acid phantom at the centre of the coil. Sixteen coronal phase-encoding steps yielded spectra from 10 mm slices (TR = heart rate, 192 averages, acquisition time approximately 20 min), using a trigger delay of 400 ms. A cosine apodisation filter was applied. The first spectrum containing signal beyond the chest wall and solely from cardiac tissue was selected. The spectrum was analysed using an AMARES time domain fit algorithm in jMRUI [23] and the ATP peak area was corrected for blood contamination [24].

### Liver and visceral fat MRI

Liver fat was assessed by ^1^H-magnetic resonance spectroscopy (TR/TR = 3,000 ms/35, 50, 75, 100, 125 or 150 ms, 3 × 3 × 3 × 3 cm voxel, SENSE torso array, one signal average and an expiration breath hold of 17 s). The water and CH_2_ resonances were analysed using the AMARES algorithm in jMRUI [23]. The T2 relaxation times of the water and CH_2_ resonances for each participant were calculated from the data by monoexponential fitting of signal intensity vs the six echo times, the signals at 35 ms were then corrected for T2 decay and the fat fraction was calculated as a percentage of the total signal from that volume [25].

**Fig. 3:**
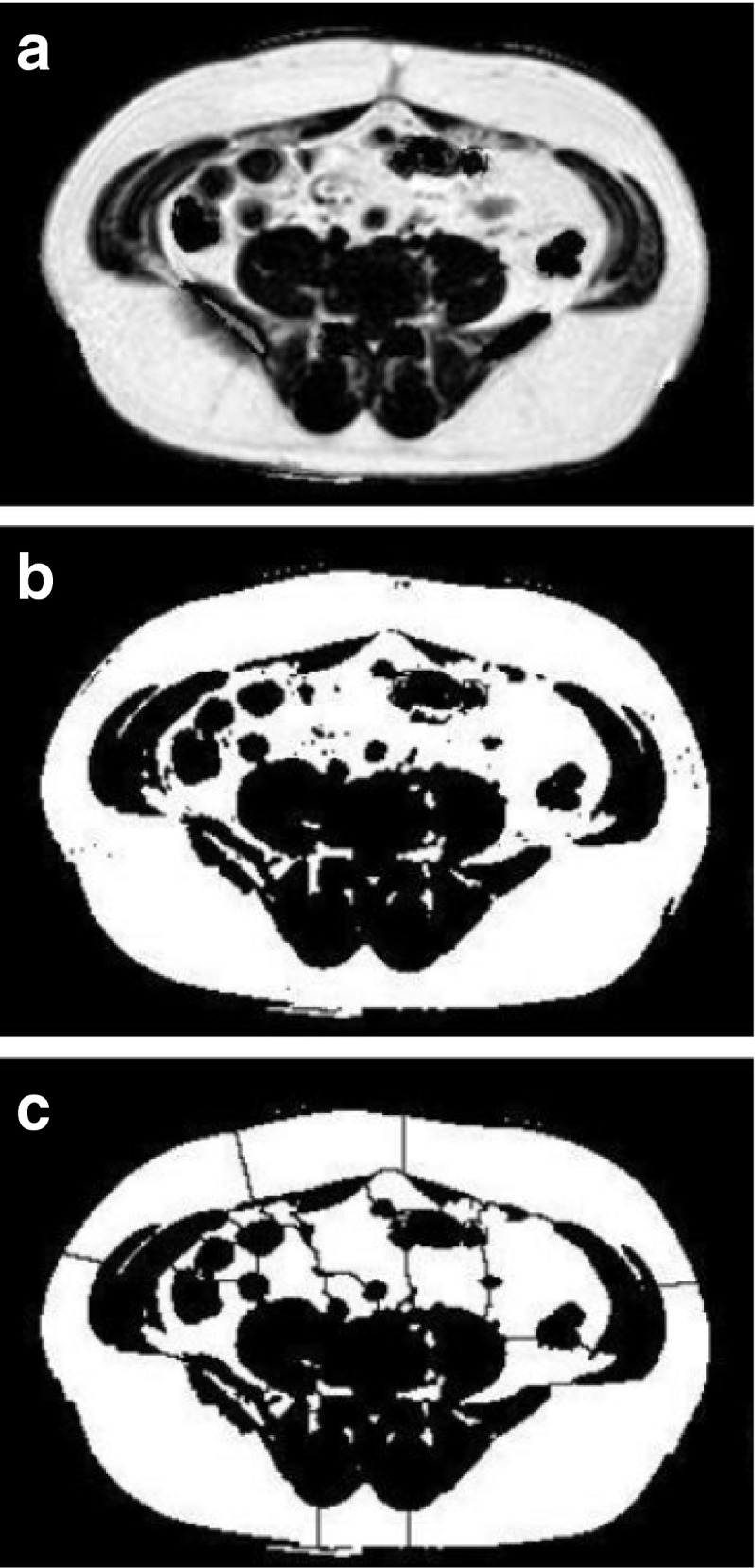
Quantifying visceral fat. (**a**) The three-point Dixon fat fraction map acquired at the L4–L5 junction. (**b**) Binary thresholding of structures containing more than 50% fat (visceral and subcutaneous fat) from those with less. Total area is evaluated. (**c**) Application of thresholding algorithm divides segmented image into chunks and separates visceral and subcutaneous fat around the boundary of the chest wall. Selection of subcutaneous fat and any external signals allows measurement of this area and subtraction from the total to yield visceral fat area

Visceral fat was estimated at the L4–L5 junction [26] using a three-point Dixon sequence (TR = 50 ms, TE = 3.45, 4.60 or 5.75 ms, number of averages = 1, flip angle = 5°, voxel size 2.5 × 2.5 mm, slice thickness 10 mm, zero filled to 1.4 × 1.4 mm, and median FOV 440 mm [range 400–480 mm to suit participant size], with 70% phase FOV) [27]. Binary gating and a watershed algorithm was used to divide the binary image into distinct areas (Fig. 3); this allowed easy separation and quantification of the subcutaneous and visceral fat areas using ImageJ [28]. Liver and visceral fat analyses were performed by a single observer blinded to group allocation.

### Glycaemic control, blood variables and body composition

After an 8 h minimum overnight fast, a 75 g OGTT was performed in which samples were drawn every 15 min and analysed for whole blood glucose (YSI 2300 Stat Plus-D, Yellow Springs Instruments, Yellow Springs, OH, USA) and plasma insulin (Mercodia Iso-Insulin ELISA, no. 10-1128-01, Mercodia, Uppsala, Sweden). Insulin resistance and beta cell function were predicted using the HOMA2 [29], and the area under the glucose curve (AUGC) was calculated using the trapezoidal rule [30]. Fasting plasma samples were analysed in an accredited clinical pathology laboratory (Department of Clinical Biochemistry, Newcastle upon Tyne Hospital NHS Foundation Trust) for alanine phosphatase (ALP), alanine transaminase (ALT), aspartate aminotransferase (AST), total cholesterol, triacylglycerols and HbA_1c_. Total cholesterol, triacylglycerols, ALP, AST and ALT were measured using a Roche P800 Modular Analyzer (Basel, Switzerland) and HbA_1c_ was measured using a TOSOH HLC-723G8 (Minato, Tokyo, Japan). Body composition was measured using air displacement plethysmography (BodPod, Life Measurement, CA, USA).

### Intervention

The HIIT group performed 36-cycle ergometry sessions over 12 weeks (3 sessions per week on non-consecutive days) at a local gym. Patients were required to perform at least 32 sessions (89% of total sessions) for inclusion in the analysis. Intensity was based on the 6–20 point Borg Rating of Perceived Exertion (RPE) [31]. Each session included a 5 min warm up in which participants would progress from RPE 9 to 13 (‘very light’ to ‘somewhat hard’), followed by five intervals, each with a pedal cadence of >80 rev/min, reaching a RPE 16–17 (‘very hard’). The final interval was then followed by a 3 min recovery cycle. Intervals lasted 2 min in week 1 and progressed by 10 s increments each week such that week 12 consisted of 3 min 50 s intervals. Three min recovery periods interspersed each interval, which consisted of 90 s passive recovery, 60 s of band-resisted upper body exercise and 30 s to prepare for the subsequent interval. The arm resistance bands (Bodymax Fitness, Clydebank, UK) were used as light recovery and involved one exercise per recovery period in the following order: face pull, horizontal push, horizontal pull and 30° push. The initial session was supervised; thereafter, participants were guided through each session by voice-recorded instructions using an iPod shuffle (Apple, CA, USA). An exercise diary was completed to monitor exercise adherence.

Apart from HIIT sessions, all study participants were instructed to continue their normal routine and care for 12 weeks and not to change medication, habitual physical activity, diet or body weight. Weekly phone calls were made to assess adherence, and habitual physical activity was assessed over 7 days pre- and post-intervention using a validated multisensory armband (Sensewear; Bodymedia, Pittsburgh, PA, USA) [32].

### Statistical analysis

Statistical power was based on the change in HbA_1c_. We selected a sample size of 12 to provide a statistical power of 80% to detect a difference of 0.6% in HbA_1c_ [33]. A sample size of 14 was used to allow for two dropouts per group. A per-protocol analysis was adopted because the intention of this study was to assess efficacy and mechanisms of change, not effectiveness. All analyses were performed using IBM SPSS Statistics software (version 19, NY, USA) and data are presented as means ± SD unless otherwise stated. Continuous data were tested for normality using the Shapiro–Wilk test. Comparisons of key baseline variables were made using independent sample *t* tests. Between-group comparisons were made using ANCOVA with the baseline value as the covariate. Within-group changes were assessed by paired-sample *t* tests or the non-parametric alternative (Wilcoxon signed-rank test) for non-normally distributed data. Adjustment for multiple comparisons was not made because of co-linearity between variables, hypothesis-driven comparisons and the increased risk of type II error following adjustment [34]. Pearson’s correlation or the non-parametric alternative (Spearman’s rank correlation) was used to calculate *r* values among body composition, metabolic and cardiac variables. *P* values <0.05 were considered statistically significant.

## Results

The groups were matched well for all baseline characteristics (Table 1). Glycaemic control was similar between groups and liver fat was above the clinically defined threshold for non-alcoholic fatty liver disease (>5%) in both groups. Adherence to intervention was good, with HIIT patients completing an average of 36 ± 0.9 sessions, and Sensewear armband activity revealed no within-group change in habitual physical activity (daily energy expenditure: HIIT 2,701 ± 299 to 2,537 ± 386, *p =* 0.129 vs control 2,548 ± 366 to 2,455 ± 166, *p =* 0.459 (calories)).

### Cardiac structure, function and energetics

**Fig. 4:**
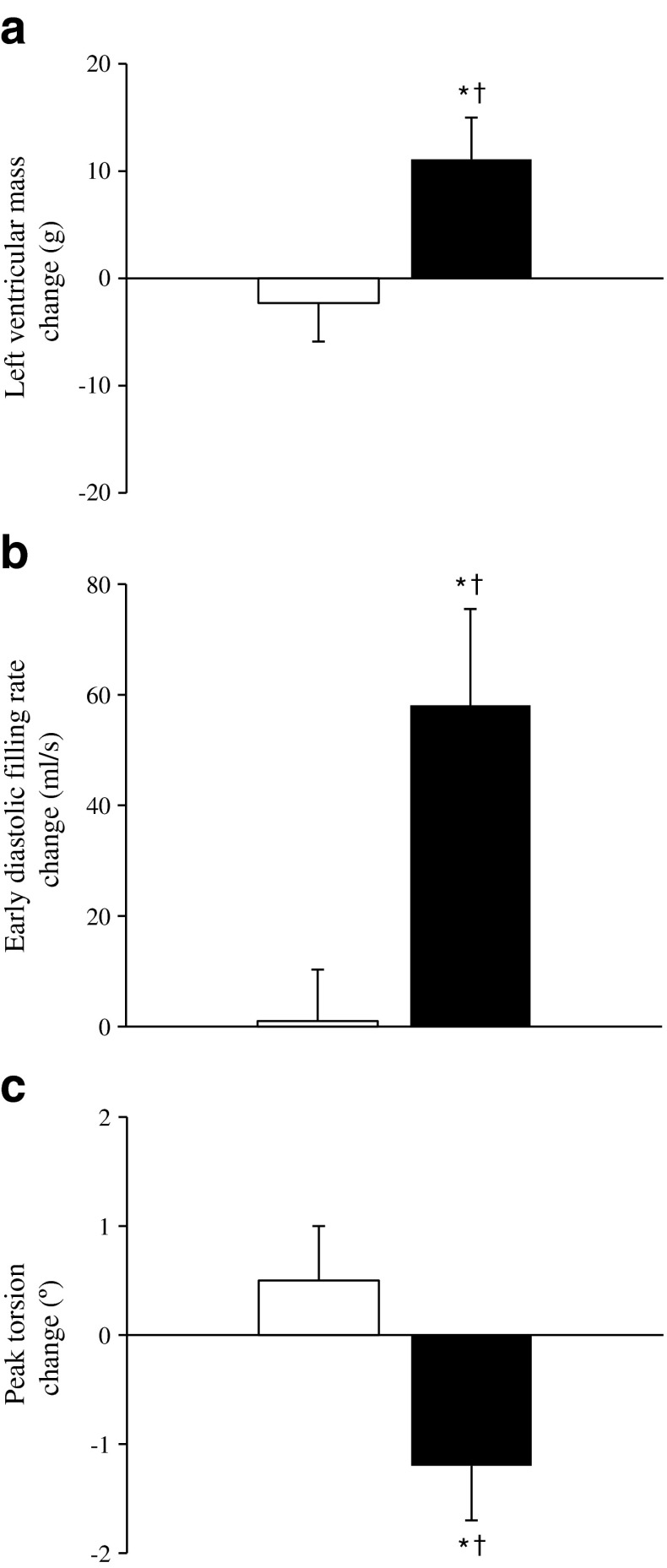
Effect of HIIT vs control on (**a**) left ventricular mass, (**b**) diastolic filling rate and (**c**) peak torsion. White bars, control; black bars, HIIT. Values are means ± SEM. *Significant difference baseline vs post-treatment (*p <* 0.05). ^†^Significant difference between-group interaction (*p <* 0.05)

HIIT induced structural cardiac changes, with a 12% relative increase in left ventricular wall mass (*p <* 0.05) and increased end-diastolic blood volume (*p <* 0.01; Fig. 4a). The exercise group also demonstrated improvements in systolic function, indicated by raised stroke volume (*p <* 0.01) and left ventricular ejection fraction (*p <* 0.05). The early diastolic filling rate increased by 24% (Fig. 4b), and within-group comparison revealed a significant increase in early filling percentage after HIIT (57 ± 9% to 60 ± 9%, *p <* 0.05; Table 2). There was a 15% relative decrease in peak torsion after exercise (8.1 ± 1.8° to 6.9 ± 1.6° vs 7.1 ± 2.2° to 7.6 ± 1.9°; *p <* 0.05; Fig. 4c); myocardial strain remained constant. The phosphocreatine (PCr)/ATP ratio did not change following HIIT (*p =* 0.115; Table 2).

**Table 2:** Effect of HIIT on cardiac structure, function and metabolism

Parameter	Control			HIIT			Adjusted between-group *p* value^b^
	Pre	Post	Within-group *p* value^a^	Pre	Post	Within-group *p* value^a^	
Cardiac structure							
Left ventricular wall mass (g)	107 ± 25	105 ± 25	0.54	104 ± 17	116 ± 20	0.02^*^	0.03^†^
Wall thickness systole (mm)	5.5 ± 1.1	6.2 ± 1.0	0.01**	6.2 ± 1.5	6.8 ± 1.1	0.07	0.54
Wall thickness diastole (mm)	9.1 ± 2.5	10.1 ± 2.5	0.02*	10.7 ± 3.1	11.5 ± 1.8	0.32	0.43
Eccentricity ratio (g/ml)	0.85 ± 0.24	0.87 ± 0.18	0.66	0.94 ± 0.28	0.96 ± 0.24	0.70	0.66
End-diastolic volume (ml)	129 ± 28	122 ± 28	0.08	118 ± 30	126 ± 30	0.01**	0.00^††^
End-systolic volume (ml)	50 ± 22	47 ± 22	0.33	42 ± 17	39 ± 13	0.25	0.76
Systolic function							
Systolic BP (mmHg)	126 ± 3	124 ± 5	0.62	123 ± 4	122 ± 4	0.66	0.99
Diastolic BP (mmHg)	84 ± 2	80 ± 2	0.07	81 ± 2	80 ± 2	0.81	0.41
Heart rate (bpm)	63 ± 7	69 ± 13	0.21	67 ± 12	66 ± 16	0.69	0.27
Stroke volume (ml)	79 ± 14	75 ± 15	0.16	76 ± 16	87 ± 19	0.00**	0.00^††^
Cardiac output (l/min)	5.0 ± 1.0	5.2 ± 1.0	0.54	5.0 ± 1.00	5.5 ± 1.0	0.07	0.31
Ejection fraction (%)	64 ± 11	63 ± 10	0.62	65 ± 8	70 ± 6	0.02*	0.03^†^
Longitudinal shortening (%)	13.1 ± 2.2	12.7 ± 2.6	0.62	12.2 ± 3.0	13.4 ± 1.8	0.28	0.39
Diastolic function							
Early filling percentage (%)	58 ± 11	59 ± 8	0.88	57 ± 9	60 ± 9	0.04*	0.45
Early diastolic filling rate (ml/s)	250 ± 44	251 ± 47	0.68	241 ± 84	299 ± 89	0.01**	0.02^†^
Late diastolic filling rate (ml/s)	310 ± 143	285 ± 60	0.68	278 ± 67	289 ± 64	0.53	0.56
Strain and torsion							
Peak endocardial circumferential strain (%)	23.1 ± 4.1	23.4 ± 4.3	0.82	25.2 ± 4.6	24.5 ± 5.1	0.61	0.82
Peak whole wall circumferential strain (%)	16.5 ± 3.1	16.0 ± 3.3	0.46	16.5 ± 3.1	16.4 ± 4.0	0.94	0.73
Peak torsion (°)	7.1 ± 2.2	7.6 ± 1.9	0.19	8.1 ± 1.8	6.9 ± 1.6	0.04*	0.04^†^
Metabolism							
PCr/ATP ratio	1.76 ± 0.51	1.72 ± 0.36	0.80	1.74 ± 0.39	2.00 ± 0.36	0.19	0.12

Data are mean ± SD

^a^Paired *t* test

^b^Adjusted for baseline value for ANCOVA

*Significant difference baseline vs post-treatment (*p <* 0.05)

**Significant difference baseline vs post-treatment (*p <* 0.01)

^†^Significant difference between-group interaction (*p <* 0.05)

^††^Significant difference between-group interaction (*p <* 0.01)

bpm, beats per minute; pre, pre-treatment; post, post-treatment

### Body composition

Within-group comparisons revealed no change in body weight after exercise. However, the 1% increase and decrease in control and HIIT, respectively, was a significant between-group interaction (*p <* 0.05; Table 3). There was no effect of HIIT on whole body fat mass but within-group comparison revealed a reduction in visceral adipose tissue (201 ± 80 cm^2^ to 181 ± 72 cm^2^, *p <* 0.05; Table 3). The change in whole body fat mass (in kg) was associated with changes in 2 h glucose (*r =* 0.46, *p =* 0.027) and HbA_1c_ (*r =* 0.60, *p =* 0.003).

**Table 3:** The effect of HIIT on body composition, blood variables and metabolic control

Parameter	Control			HIIT			Adjusted between-group *p* value^b^
	Pre	Post	Within-group *p* value^a^	Pre	Post	Within-group *p* value^a^	
Body composition							
Weight (kg)	90 ± 9	91 ± 10	0.06	90 ± 15	89 ± 15	0.09	0.02^†^
Fat mass (kg)	35.6 ± 10.9	36.0 ± 11.3	0.36	31.9 ± 9.3	30.8 ± 10.2	0.09	0.08
Fat free mass (kg)	54.3 ± 5.9	54.7 ± 5.7	0.28	57.7 ± 9.0	58.2 ± 8.9	0.34	0.72
Visceral adipose tissue (cm^2^)	159 ± 58	156 ± 49	0.21	201 ± 80	181 ± 72	0.04*	0.08
Liver fat (%)	7.1 ± 6.8	7.7 ± 6.9	0.12	6.9 ± 6.9	4.2 ± 3.6	0.06	0.01^††^
Blood variables							
ALT (U/l)	34 ± 16	33 ± 14	0.82	36 ± 11	30 ± 10	0.02*	0.14
AST (U/l)	27.6 ± 10.4	26.5 ± 8.8	0.63	27 ± 7	24 ± 6	0.02*	0.25
ALP (U/l)	59.2 ± 16.8	61.2 ± 17.5	0.09	66 ± 17	63 ± 16	0.10	0.03^†^
Total cholesterol (mmol/l)	4.5 ± 0.9	4.6 ± 0.9	0.62	4.0 ± 1.0	4.5 ± 1.1	0.15	0.77
Triacylglycerol (mmol/l)	1.1 ± 0.4	1.2 ± 0.4	0.12	1.1 ± 0.3	1.2 ± 0.4	0.28	0.87
Metabolic control							
HbA_1c_ (%)	7.2 ± 0.5	7.4 ± 0.7	0.07	7.1 ± 1.0	6.8 ± 0.9	0.10	0.02^†^
HbA_1c_ (mmol/mol)	54.9 ± 5.9	57.0 ± 7.5	0.06	54.5 ± 10.6	51.3 ± 10.2	0.09	
Fasting glucose (mmol/l)	7.0 ± 1.0	7.6 ± 1.4	0.03*	6.8 ± 1.6	6.8 ± 1.6	0.87	0.15
Fasting insulin (pmol/l)	81.5 ± 46.4	88 ± 39.5	0.42	65.5 ± 39.5	65.5 ± 32.8	0.88	0.22
2 h glucose (mmol/l)	11.7 ± 3.1	12.9 ± 2.7	0.01**	12.5 ± 3.1	11.7 ± 3.1	0.22	0.02^†^
2 h AUGC	1,366 ± 66	1,544 ± 86	0.01**	1,395 ± 81	1,399 ± 87	0.94	0.02^†^
HOMA-IR	1.6 ± 0.9	1.8 ± 0.8	0.40	1.3 ± 0.8	1.4 ± 0.6	0.94	0.19
HOMA2-β	67.8 ± 31.4	67.0 ± 37.3	0.79	68.9 ± 48.6	70.9 ± 49.0	1.00	0.76
HOMA2-S	76.1 ± 33.0	67.3 ± 29.5	0.25	101.7 ± 48.1	98.2 ± 53.8	0.88	0.39

Data are mean ± SD

^a^Paired *t* test

^b^Adjusted for baseline value for ANCOVA

*Significant difference baseline vs post-treatment (*p <* 0.05)

**Significant difference baseline vs post-treatment (*p <* 0.01)

^†^Significant difference between-group interaction (*p <* 0.05)

^††^Significant difference between-group interaction (*p <* 0.01)

Pre, pre-treatment; post, post-treatment

### Liver fat and enzymes

HIIT elicited a 39% relative reduction in liver fat (6.9 ± 6.9% to 4.2 ± 3.6%, *p <* 0.05) so that four patients in the exercise group moved from having clinically significant liver fat to within ‘normal’ limits (<5%, Table 3). There was a significant between-group interaction for HIIT and liver fat (*p <* 0.05; Table 3), accompanied by within-group changes in ALT and AST (*p <* 0.5; Table 3), both markers of liver damage. Change in liver fat correlated with change in fasting glucose (*r =* 0.45, *p =* 0.030), 2 h glucose (*r =* 0.57, *p =* 0.004) and HbA_1c_ (*r =* 0.70, *p =* 0.000).

### Glycaemic control

HIIT had no impact on fasting glucose (6.8 ± 1.6 mmol/l to 6.8 ± 1.6 mmol/l, *p =* 0.866) or fasting insulin (65.5 ± 39.5 pmol/l to 65.5 ± 32.8 pmol/l, *p =* 0.875); however, between-group comparisons revealed improvements in HbA_1c_, 2 h glucose and AUGC (*p <* 0.05; Table 3). There was no improvement in insulin sensitivity (HOMA2 of insulin resistance [HOMA2-IR] and HOMA2 of insulin sensitivity [HOMA2-%S]) or beta cell function (HOMA2 of beta cell function [HOMA2-β]).

## Discussion

This is the first study to examine the effects of HIIT on cardiac structure and function, regional fat deposition, and glycaemic control in people with type 2 diabetes. The main findings were that a 12 week HIIT programme increased left ventricular wall mass and end-diastolic blood volume, improved systolic and diastolic function, reduced peak torsion, and decreased liver fat. Therefore, HIIT was an effective strategy to reverse cardiac dysfunction and reduce liver fat in this patient group and was accompanied by modest improvements in glycaemic control.

### Cardiac changes

Left ventricular wall mass and end-diastolic blood volume increased after 12 weeks of HIIT. This ‘physiological hypertrophy’ is a known effect of exercise but should not be confused with ‘pathological hypertrophy’, as seen in those with type 2 diabetes [4, 35]. An increase in cardiomyocyte size and protein synthesis is observed during physiological and pathological hypertrophy in response to either growth or stress signals, respectively [35]. These conditions differ in that only pathological hypertrophy is characterised by collagen accumulation and increased wall thickness, which compromises end-diastolic blood volume and is an independent predictor of cardiovascular death [36]. This is the first study using MRI to show that HIIT can stimulate positive cardiac remodelling.

The increase in stroke volume and ejection fraction is important because those with type 2 diabetes have reduced cardiac contractile capabilities [4]. Cardiomyocyte responses to high intensity exercise training in animal models have demonstrated improvements in the maximal extent of shortening, contraction and relaxation rates, with twice the improvement seen after training at 85–90% compared with training at 65–70% $\dot{V}{\mathrm{O}}_{2\mathrm{peak}}$ [37]. The increased myofilament sensitivity to calcium and the faster rise and diastolic decay of the calcium transient underpins these changes [37].

The early filling rate increased by 24%, suggesting that the myocardium is more compliant and quicker to relax following HIIT. Diastolic dysfunction is the most widely reported malfunction of the diabetic heart and an independent predictor of mortality [5, 7, 38], thus emphasising the clinical relevance of these findings. One study using echocardiography demonstrated diastolic improvements following 12 weeks of HIIT [14] and a longer term intervention using moderate exercise demonstrated improvements only when exercise was performed in the vigorous zone [39].

The decrease in torsion after HIIT provides, for the first time, evidence that exercise can be used to reverse the raised cardiac torsion observed in type 2 diabetes [8]. Cardiac torsion is a normal feature of contraction in a healthy heart and reflects the dominance of epicardial fibres over endocardial fibres [40]. Raised torsion in type 2 diabetes is a consequence of impaired contraction of endocardial fibres, which are less able to counteract this twisting motion [40]. These results indicate that endocardial damage and potential perfusion deficits at the endocardium can be improved with HIIT. No change was observed in peak endocardial and peak whole wall circumferential strain because the relative contribution of fibres across the myocardial wall remained constant, as reflected by the maintained eccentricity ratio.

HIIT stimulated improvements in cardiac structure and function independent of changes in cardiac metabolism. It was previously suggested that defects in cardiac metabolism underlie cardiac abnormalities seen in type 2 diabetes [7]; however, the decrease in PCr/ATP ratio in type 2 diabetes most likely reflects changes in substrate supply to the heart rather than an underlying metabolic defect in the myocardium [6].

### Metabolic changes

These data reveal for the first time that HIIT can reduce liver and visceral fat in type 2 diabetes, which is clinically important because both fat depots play a key pathogenic role in this chronic disease [16, 17]. To our knowledge, this is the greatest reduction in liver fat to be reported following exercise in type 2 diabetes.

Despite this, fasting blood glucose did not change and, in line with data in healthy adults and obese women [41, 42], the results demonstrated no impact of HIIT on central insulin sensitivity in adults with type 2 diabetes. These results differ from those obtained after the very low energy diet (2,512 kJ [600 kcal]), which led to a 30% relative reduction in liver fat and normalisation of fasting blood glucose after just 7 days [43]. In the present study, however, there was large inter-individual variation in liver fat changes after HIIT, which may explain the absence of change in fasting blood glucose within this small sample. Indeed, those who lost the greatest liver fat had the largest reductions in fasting glucose, as reflected in the significant correlation.

It has been reported that HIIT acutely improves peripheral insulin sensitivity when measured within 72 h of the last exercise bout, attributable to rapid glycogen breakdown and subsequent re-synthesis [41]. The two studies which have measured postprandial response to HIIT in type 2 diabetes used continuous blood glucose measurements under standard dietary conditions [44, 45]. Dietary intake was not standardised in the present study, which may explain the lack of within-group improvement in 2 h glucose or HOMA-IR.

Within-group analysis also revealed no significant impact of HIIT on HbA_1c_. The significant between-group interactions most likely reflect a worsening of glycaemic control in the control group. HbA_1c_ was reduced by around 0.3% following HIIT, which is less than the reported mean effect (0.6% reduction) of exercise interventions [33]. That being said, the greatest improvements in 2 h glucose and HbA_1c_ occurred in those who lost the largest amount of whole body fat mass and liver fat. As for fasting blood glucose, we speculate that 2 h glucose and HbA_1c_ within-group changes failed to reach significance because of the variation in liver fat reductions following HIIT.

This study questions the impact of HIIT on glycaemic control in adults with type 2 diabetes, but also confirms the importance of ectopic fat. Patients were required to maintain their weight during the HIIT programme, which may have compromised any improvements in glucose control. Interventions to target weight loss and ectopic fat may be most beneficial for glycaemic improvements. Despite this, these data highlight the positive impact of exercise on cardiac health, which may be improved further when accompanied by weight loss.

This study is not without limitation. The physiological mechanisms underlying the cardiac adaptations could not be elucidated with the MRI techniques used. Although magnetic resonance methods for quantifying ATP flux in the human heart by cardiac spectroscopy have been demonstrated in heart failure [46], they remain time-consuming and prone to variability. The PCr/ATP ratio can be measured in a reasonable period of time and has been demonstrated to be impaired in type 2 diabetes [47]. Using the RPE as a guide for exercise intensity (rather than an objective measure) may have limited the accuracy of the training intensity; however the RPE has been found to be an accurate predictor of exercise intensity in diabetes [48]. Finally, dietary monitoring was not adopted throughout the intervention.

In summary, this study demonstrates for the first time improvements in cardiac structure and function in patients with type 2 diabetes following a HIIT programme. These changes were accompanied by modest improvements in glycaemic control. HIIT elicited the greatest reduction in liver fat to be recorded following an exercise intervention in type 2 diabetes, demonstrating that this type of exercise is effective at targeting fat depots which play a role in the aetiology of this chronic condition. Although the direct benefits of HIIT to glycaemic control remain uncertain, HIIT is a potential therapy to moderate cardiac risk and reduce liver fat in type 2 diabetes and should be considered by clinicians alongside other regimens to improve glycaemic control.

## Abbreviations

ALP: Alanine phosphatase
ALT: Alanine transaminase
AST: Aspartate aminotransferase
AUGC: Area under the glucose curve
HIIT: High intensity intermittent training
PCr: Phosphocreatine
RPE: Rating of perceived exertion
$\dot{V}{\mathrm{O}}_{2\mathrm{peak}}$: Peak oxygen consumption
